# Validation of Broth Macrodilution Volatilization Method for Testing of Essential Oils in Liquid and Vapor Phase: Chemical Composition, Cytotoxicity, and Antibacterial Effect of Indian Medicinal Plants against Pneumonia-Causing Pathogens

**DOI:** 10.3390/molecules28124625

**Published:** 2023-06-07

**Authors:** Aishwarya Chaure, Marketa Houdkova, Julien Antih, Klara Urbanova, Ivo Doskocil, Mukund Lal Naik, Khageshwar Singh Patel, Ladislav Kokoska

**Affiliations:** 1 Department of Crop Sciences and Agroforestry, Faculty of Tropical AgriSciences, Czech University of Life Sciences Prague, 16500 Prague, Czech Republic; chaure@ftz.czu.cz (A.C.); houdkovam@ftz.czu.cz (M.H.); antih@ftz.czu.cz (J.A.); 2 Department of Sustainable Technologies, Faculty of Tropical AgriSciences, Czech University of Life Sciences Prague, 16500 Prague, Czech Republic; urbanovak@ftz.czu.cz; 3 Department of Microbiology, Nutrition and Dietetics, Faculty of Agrobiology, Food and Natural Resources, Czech University of Life Sciences Prague, 16500 Prague, Czech Republic; doskocil@af.czu.cz; 4 National Center for Natural Resources, Pt. Ravishankar Shukla University, Raipur 492010, India; mlnaik1943@gmail.com; 5 Department of Applied Sciences, Amity University, Manth (Kharora), State Highway 9, Raipur 493225, India; patelkhageshwarsingh@gmail.com

**Keywords:** antimicrobial activity, *Cymbopogon citratus*, *Cyperus scariosus*, GC/MS, headspace analysis, macrodilution, MTT assay, respiratory infections, *Trachyspermum ammi*, vapor phase, volatiles

## Abstract

Essential oils (EOs) have great potential in inhalation therapy for the treatment of respiratory infections. However, innovative methods for evaluation of antimicrobial activity of their vapors are still needed. The current study reports validation of the broth macrodilution volatilization method for assessment of the antibacterial properties of EOs and shows the growth-inhibitory effect of Indian medicinal plants against pneumonia-causing bacteria in liquid and vapor phase. Among all samples tested, *Trachyspermum ammi* EO exhibits the strongest antibacterial effect against *Haemophilus influenzae*, with minimum inhibitory concentrations of 128 and 256 µg/mL in the liquid and vapor phases, respectively. Furthermore, *Cyperus scariosus* EO is found to be nontoxic to normal lung fibroblasts assessed by modified thiazolyl blue tetrazolium bromide assay. Chemical analysis performed using gas chromatography–mass spectrometry identified α-citral, cyperotundone, and thymol as the main constituents of *Cymbopogon citratus*, *C. scariosus*, and *T. ammi* EOs, respectively. In addition, β-cymene is identified as the major compound of *T. ammi* EO vapors when analyzed using solid-phase microextraction and gas-tight syringe sampling techniques. This study demonstrates the validity of the broth macrodilution volatilization method for antimicrobial screening of volatile compounds in the vapor phase and suggests the therapeutic potential of Indian medicinal plants in inhalation therapy.

## 1. Introduction

Pneumonia is defined as acute infection of the lung parenchyma [1], typically caused by bacterial species including gram-positive *Staphylococcus aureus*, *Streptococcus pneumoniae*, *Streptococcus pyogenes*, and gram-negative *Haemophilus influenzae* [2]. It is a major health problem associated with high morbidity and mortality, especially in developing countries, where vulnerable populations such as children of <5 years of age and older adults with prior chronic conditions are at great risk [3]. In the year 2020, India alone contributed 23% of the global pneumonia burden with case fatality rates between 14 and 30% [4]. The general approach in the treatment of pneumonia includes the timely administration of appropriate antibiotic therapy. Recently, nebulized antibiotics have become preferred over intravenous and oral therapies, as they allow high pulmonary efficacy and minimal systemic side effects [5]. Antibiotics currently marketed for inhalation treatment of chronic *Pseudomonas aeruginosa* infections include tobramycin, colistin, and aztreonam. Although both the U.S. Food and Drug Administration (FDA) and European Medicines Agency have approved their use for cystic fibrosis, they have not been approved in other disease areas because of a lack of supportive clinical trial evidence [6]. Moreover, other problems are associated with the distribution and deposition of aerosol particles, whose size plays a major role in efficient delivery. For example, larger particles preferably accumulate in the oropharyngeal area, while smaller particles deposit in the lower airways, which alters drug delivery, as small particles carry fewer active substances. In addition, patient-related factors such as age, physical capability, disease severity, and the cognitive ability of the patient to perform specific inhalation affect drug delivery [7].

Essential oils (EOs) can be promising sources for the development of new inhalation preparations, as the physical property of being volatile at room temperature enables them to freely distribute among the lung tissues [8]. Therefore, in contrast with nebulized antibiotics, EO vapors allow easy inhalation and uniform distribution of active substances in the upper and lower parts of the respiratory tract [9]. Furthermore, due to their antimicrobial and anti-inflammatory potency in the vapor phase, they offer effective treatment for various respiratory infections. In the last few years, several inhalation devices using a combination of different EOs have been developed and patented. For example, adhesive inhalation antiviral patches (Axogen Inc., Alachua, FL, USA), which contain safe and effective amounts of EOs obtained from plant species such as *Cinnamomum verum* J. Presl, *Citrus limon* (L.) Osbeck, *Gaultheria procumbens* L., *Matricaria recutita* L., *Mentha* × *piperita* L., *Salvia sclarea* L., *Syzygium aromaticum* (L.) Merr. & L.M. Perry, and *Zingiber officinale* Roscoe are usually placed near the nasal pathway with the application of an appropriate mask, which prevents the entry of various respiratory-infection-causing pathogens [10]. In addition, various herbal products based on EOs, e.g., GeloMyrtol (G. Pohl-Boskamp, Hohenlockstedt, Germany) and Bronchipret (Bionorica, Neumarkt, Germany), are recommended for the treatment of acute and chronic bronchitis [11]. EOs are very complex natural mixtures of compounds that work in synergy to provide various medicinal properties. They are mainly composed of aromatic and aliphatic compounds, hydrocarbon terpenes (isoprenes), and terpenoids (terpene hydrocarbons such as phenols, alcohols, ketones, aldehydes, acids, esters, and ethers) [12]. Especially the phenolic monoterpenoids, such as carvacrol and thymol, have been reported to exhibit a strong antibacterial effect in the vapor phase against pathogens that cause pneumonia [13]. The bioactivity of EOs depends greatly on their chemical composition, which makes the chemical characterization of volatile components an important work [14]. Nowadays, static headspace extraction, coupled with the gas chromatography–mass spectrometry (S-HS-GC/MS) technique, is commonly used to investigate EO vapors. It is a simple, rapid, and solventless technique that requires a small amount of sample and allows the analysis of highly volatile compounds [15]. Correspondingly, several studies have explored the chemical composition of EO vapors with the aim to find the most prominent volatile compounds responsible for antibacterial activity in the vapor phase using S-HS-GC/MS. For example, a study conducted by Schweitzer et al. [16] has explored the chemical composition of *C. citratus* EO vapors using solid-phase microextraction followed by GC/MS. Using this technique, citral, α-terpineol, γ-cadinene, and calamenene have been found to be the major components of EO vapors that are attributed to their antibacterial and antifungal activities. In another study, chemical investigation of *T. ammi* EO has been conducted using headspace–solid phase microextraction (HS-SPME) coupled with GC/MS, where γ-terpinene, *p*-cymene, and thymol were found to be major compounds [17].

In terms of plant diversity, 1500 species have been reported to have medicinal uses in India [18], many of which have not been phytochemically and pharmacologically explored [19]. Indian Ayurvedic medicine has a long tradition in the management of various respiratory diseases using poly-herbal preparations and multicomponent therapeutics. Several pharmaceutical companies are manufacturing and marketing different Ayurvedic formulations [20]. For example, Bresol-NS (Himalaya, Bengaluru, India), a nasal spray with a combination of *Coleus aromaticus* Benth., *Eucalyptus globulus* Labill., and *Glycyrrhiza glabra* L. extracts, helps relieve nasal congestion caused by upper respiratory tract infections. Administration of drugs through the nasal route is an integral part of Ayurvedic practices such as *dhumpana* (medicinal smoking), *nasya* (nasal administration of therapeutic oil), and *dhupanartha* (herbal fumigation). In addition, ethnobotanical evidence of using vapor-based medicines has been well-documented in northeast India. For example, in Manipur, the people of the Meitei community use leaves of *Phlogacanthus thyrsiflorus* Nees for steam inhalation in the treatment of pneumonia. Similarly, the stems of *Clerodendrum indicum* (L.) Kuntze and *Rotheca serrata* (L.) Steane & Mabb. are inhaled via smoke for treatment of acute bronchitis [21]. Among various plant species used in Indian traditional medicine for the treatment of respiratory infections, *Cymbopogon citratus* (DC.) Stapf and *Trachyspermum ammi* (L.) Sprague and certain species of genera *Cyperus* (e.g., *Cyperus rotundus* L.) are common ingredients for vapor-based medicines [22,23,24]. In correspondence with their traditional use, the antibacterial potential of EO vapors of the above-mentioned species has been previously reported in several in vitro experiments. For example, a study performed by Inouye et al. [25] assessed the growth-inhibitory effects of *C. citratus* EO vapors against respiratory tract pathogens such as *H. influenzae*, *S. aureus*, *S. pneumoniae*, and *S. pyogenes* using the airtight box disc volatilization method. In another study, *T. ammi* EO vapors exhibited a growth-inhibitory effect against bovine respiratory bacterial pathogens using an agar plug vapor-phase assay [26]. However, the antibacterial potential of *T. ammi* and *Cyperus scariosus* R.Br. EO vapors against pneumonia-causing bacteria has not been fully explored yet. Moreover, the methods used for examining the antimicrobial effect of the above-mentioned EO vapors provided mainly qualitative results, or in the case of quantitative methods, the results were expressed in different ways, e.g., as a concentration of volatile agent per volume of air [25], and interpretation of results, including minimum inhibitory concentration (MIC) values, was varying greatly [27]. Although EOs and their compounds are usually considered as safe, and they are registered as generally recognized as safe (GRAS) products by the U.S. FDA, toxicological evaluations are still necessary to know the risks associated with direct inhalation of EOs or their constituents on lung tissues [28]. There are several studies describing the low cytotoxicity of *C. citratus* EO when tested in vitro on human non-cancer fibroblasts [29]; however, there are no data on toxicity of *T. ammi* and *C. scariosus* EOs to lung cells.

The current study reports an assessment of the antibacterial properties of selected EOs from Indian medicinal plants, namely *C. citratus*, *C. scariosus*, and *T. ammi*, with the aim to validate the newly developed broth macrodilution volatilization method for simultaneous testing of EOs in liquid and vapor phase against the bacteria causing pneumonia. Additionally, their major chemical constituents responsible for growth-inhibitory effects were identified using GC/MS, and the chemical profile of EO vapors was determined by a time series of headspace analyses including both HS-SPME and headspace–gas-tight syringe (HS-GTS) sampling techniques. Moreover, the cytotoxicity of EOs against normal lung fibroblasts using a modified thiazolyl blue tetrazolium bromide (MTT) assay was evaluated in this study.

## 2. Results

### 2.1. Antibacterial Activity and Cytotoxicity

The results showed that all EOs produced a certain level of in vitro growth-inhibitory activity against the pneumonia-causing bacterial strains included in this study. The effectiveness of EOs varied substantially in the MIC values ranging from 128 to 1024 µg/mL in the liquid phase and being equal or greater than 256 µg/mL in the vapor phase. Among all samples tested, *T. ammi* EO was found to be the most active with respective MICs of 128 and 256 µg/mL in the liquid and vapor phases against *H. influenzae,* followed by C. *citratus* EO with growth-inhibitory effect against *H*. *influenzae* with MIC 256 µg/mL in both phases. The *C. scariosus* EO exhibited weak antibacterial effect with MIC 1024 µg/mL in the liquid phase against all bacteria tested, while vapors of this EO were active against *H. influenzae* (MIC 1024 µg/mL) only. In general, a higher activity was observed in the liquid than in the vapor phase, and *H. influenzae* was the most susceptible bacterium to the EOs assayed. Complete results on the antibacterial activity of Indian EOs against respiratory pathogens in both liquid and vapor phases are shown in Table 1.

**Figure 1:**
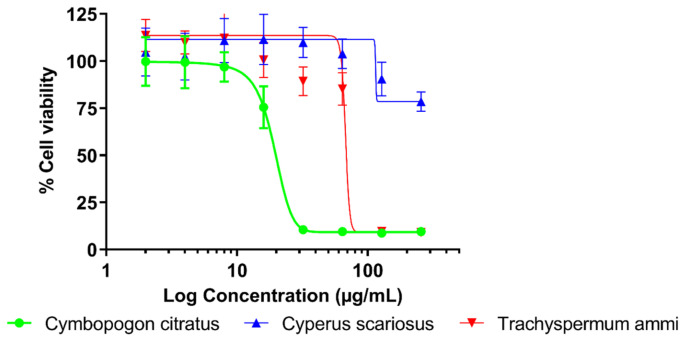
Cytotoxic activity of eight twofold serially diluted concentrations (2–256 µg/mL) of *Cymbopogon citratus*, *Cyperus scariosus* and *Trachyspermum ammi* EOs to lung fibroblast cells MRC-5 tested by MTT assay performed in microtiter plates sealed with vapor barrier EVA Capmat.

The results of a cytotoxicity assay showed that the values of half maximal inhibitory concentration (IC_50_) varied substantially for all EOs tested. *C. scariosus* EO was evaluated as nontoxic (IC_50_ > 258 µg/mL), whereas both *C. citratus* and *T. ammi* EOs were found to be moderately toxic with respective IC_50_ values of 19.63 and 82.04 µg/mL. Similarly, the highest 80% inhibitory concentration of proliferation (IC_80_) values were calculated for *C. scariosus* (IC_80_ ≥ 258 µg/mL). According to the WHO [30], the EOs were classified as nontoxic (*C. scariosus*) and moderately toxic (*C. citratus* and *T. ammi*). The therapeutic index (TI) calculated for comparison of the antibacterial and cytotoxic effects indicates safety of the *C. scariosus* EO (TI > 0.252). The detailed results of the cytotoxicity of EOs to lung fibroblasts are shown in Table 2 with a graphical presentation in Figure 1.

### 2.2. Chemical Composition

Based on GC/MS analysis of EOs from *C. citratus* (aerial part), *C. scariosus* (rhizomes), and *T. ammi* (seeds), a total of 17, 28, and 9 components were identified using HP-5MS column, which represents 99.62, 91.48, and 99.47% of their total respective contents. Similarly, using the DB-HeavyWAX column, 15, 36, and 13 components were determined, which constituted 68.77, 77.17, and 84.26% of the EOs, respectively. In total, 21 compounds were identified in the *C. citratus* EO, 40 compounds in *C. scariosus* EO, and 15 in *T. ammi* EO. The analysis showed that in *C. citratus* and *T. ammi* EOs, monoterpene hydrocarbons and oxygenated monoterpenoids were the predominated groups of chemicals, whereas sesquiterpene hydrocarbons and oxygenated sesquiterpenoids were major classes of compounds in *C. scariosus* EO. For both the columns, in *C. citratus* EO, α- and β-citral were the most abundant compounds with peak areas of (HP-5MS = 48.9 and 35.8%) and (DB-HeavyWAX = 24.3 and 33.2%), respectively. Caryophyllene oxide was the third abundant compound in the EO (~3%). In the case of *C. scariosus* EO, cyperotundone was the major component with peak areas of (29.1 and 28.9%), followed by caryophyllene oxide (19.8 and 17.54%) and cyperene with (9.9 and 8.5%), when measured using HP-5MS/DB-HeavyWAX columns, respectively. Thymol was the most predominant substance reported for both the columns with 51.2 and 45.8% in *T. ammi* EO, followed by β-cymene (22.6 and 17.1%) and γ-terpinene (21.5 and 17.6%) for HP-5MS/DB-HeavyWAX columns, subsequently. The complete chemical profiles of *C. citratus*, *C. scariosus*, and *T. ammi* EOs are provided in Table 3, Table 4 and Table 5, respectively.

### 2.3. Chemical Composition of T. ammi EO Vapors

In the current study, the composition of headspace above the mixture of *T. ammi* EO and Mueller–Hinton broth (MHB) has been carried out using HS-SPME and HS-GTS in a time series every 3 h during a 12 h period using the HP-5MS column. Using HS-SPME extraction, a total of six volatile compounds were identified in the EO sample, which represented 97.25% of their respective total constituents. When using the HS-GTS extraction method, a lower number (five) of compounds was detected, which accounted for 93.4% of their total content. Regardless of the extraction method used, monoterpenes were the most predominant chemical groups of volatile compounds identified in the headspace. Using the HS-SPME extraction method, β-cymene was the most abundant constituent of the headspace of *T. ammi* EO. The content of β-cymene gradually decreased during the whole experiment with the peak area value ranging from 49% (time—0 h) to 43% (time—12 h). Similarly, a slight decrease in the concentration of γ-terpinene, the second-most abounding compound in the sample, was observed during overtime incubation from 39 to 31%. In contrast, the concentration of the thymol increased in the vapor over the time from 4.9 to 12%. In the case of HS-GTS extraction, β-cymene, γ-terpinene, and β-pinene were detected as the predominant compounds. For β-cymene and γ-terpinene, a steady decrease in the concentrations was observed during the experiment, ranging from 52 to 45% and 35 to 28%, respectively. Although the concentrations of both most-abundant compounds are nearly similar to those obtained using the HS-SPME method, the third-most abounding compound differed for the HS-GTS extraction method, β-pinene being detected with peak area values ranging from 2 to 6.5%. Interestingly, the chemical analysis showed that content of thymol detected by HS-SPME extraction was much higher in comparison with HS-GTS extraction (nearly 10%). Apart from this, no discrepancies for either sampling method were observed, and there were no significant changes in the chemical composition in the vapor of *T. ammi* EO over time. A complete chemical profile of *T. ammi* EO vapors is provided in Table 6.

## 3. Discussion

*T. ammi* is a medicinal plant highly valued in traditional Ayurvedic medicine [23]. Its EO has been reported to have pivotal antibacterial properties against foodborne and spoilage bacteria; however, only a few studies have reported its effect against pneumonia-causing pathogens [33,34]. The current study suggests the significant antibacterial activity of *T. ammi* EO against the targeted bacteria in both liquid and vapor phases. The observed endpoints for the liquid phase correspond well with a previously published study reporting the MIC of *T. ammi* EO against *S. aureus* at 500 µg/mL [35] and within a three-dilution MIC range for *S. pneumoniae* (MIC = 250 µg/mL) [36]. According to our best knowledge, the antibacterial activity of *T. ammi* EO vapors was described for the first time in this study. Considering the toxicity, the current study reports that *T. ammi* EO is moderately cytotoxic to lung fibroblasts. This result corresponds with an in vivo study published by Vazirian et al. [37], who observed mild oral toxicity of the EO in a rat model. Regarding inhalation toxicity, the sources of information on the safety of EO vapors are scarce. However, data on its predominant compound, thymol, might suggest its possible inhalation safety. The European Chemicals Agency reported thymol to be nontoxic to mice when they are exposed to the chemical via inhalation of its vapor for 2 h with a lethal dose 50% (LD_50_) of 7.57 mg/L [38]. Moreover, another study published by Xie et al. [39] reported no evidence of chronic toxicity of thymol through inhalation in a mouse model. Nevertheless, further experiments on their in vivo inhalation toxicity are necessary to determine the safety of *T. ammi* EO. The biological properties of *T. ammi* EO are attributed to its chemical composition, which has already been studied. The chromatographic profile obtained in the current study corresponds with previously published reports identifying thymol as the main component (ranging from 54.32 to 67.4%), β-cymene as the second-most abundant compound (ranging from 17.9 to 21.74%, respectively), and γ-terpinene as third-most abundant component (ranging from 11.3 to 19.38%) [35,40]. Moreover, research led by Modareskia et al. [41] investigated the bioactive potential of different populations of *T. ammi* EO in liquid phase and reported that the antibacterial effects are mainly due to the presence of phenolic and hydrocarbon monoterpenes such as thymol, *p*-cymene, and γ-terpinene. β-Cymene, γ-terpinene, and thymol were identified as the key constituents of vapors of *T. ammi* EO in the sample obtained using the HS-SPME technique. This is well-corresponding with the results of Liu et al. [17], who reported γ-terpinene (26.21%), *p*-cymene (23.58%), and thymol (20.02%) as the major components of *T. ammi* EO vapor analysis by HS-SPME. Because the EO samples for headspace analysis performed in this study were prepared by dissolving them in microbiological growth medium, variances in the concentration of *γ*-terpinene can be attributed to the different experimental conditions [42]. Our paper identifying β-cymene, γ-terpinene, and β-pinene as predominant components of *T. ammi* EO vapors is the first report on its analysis using the HS-GTS method. When comparing both sampling techniques, the present study reports disparities with thymol concentration. In comparison with the HS-SPME method, the analysis of the sample collected by the HS-GTS technique showed a lower amount of thymol. The possible explanation may be that it was due to the sensitivity and affinity of SPME fibers toward certain volatile components [43]. In contrast, the HS-GTS technique is less selective but provides a precise and, perhaps, closer assessment of the real distribution of volatile compounds in vials [44]. These findings are consistent with the data published by Antih et al. [42], who performed *Thymus vulgaris* L. EO headspace analysis using similar experimental conditions. The antibacterial potential of volatile monoterpene hydrocarbons (*p*-cymene and γ-terpinene) and phenolic monoterpene (thymol) are well-known; for instance, the bioactivity of thymol in the vapor phase against respiratory tract bacteria such as *H. influenzae*, *S. aureus*, and *S. pneumoniae* has been previously published [13]. As mentioned before, the antibacterial activity of *T. ammi* EO and its vapors is mostly due to its composition; particularly, the presence of hydrocarbon and phenolic monoterpenes in high concentrations are related to the growth-inhibitory effect. Moreover, the current study also reports higher activity in the broth medium as compared to the vapor phase. This can be related to data obtained from the headspace analysis, where thymol, which is regarded as the main antimicrobial constituent, was detected in lower amounts in the EO vapors. The hydrophobic nature of thymol worsening its solubility in water-based media and, subsequently, reducing its volatility, can be a possible reason of lower antimicrobial activity [45].

*C. citratus* EO is extensively used in Ayurvedic medicine as a folk remedy for coughs, flu, and pneumonia [46]. Previously published studies have reported the antibacterial and anti-fungal properties of *C. citratus* EO [47,48]. As a result of the modified agar dilution method, Inouye et al. [48] observed a moderate growth-inhibitory effect of *C. citratus* EO against *S. pyogenes* (MIC = 400 µg/mL), *H. influenzae*, and *S. pneumoniae* (MIC = 800 µg/mL). In addition, *C. citratus* EO vapors were reported to be more efficient against *H. influenzae* as compared to *S. aureus*, *S. pneumoniae*, and *S. pyogenes*. Despite certain variances in the MIC values caused probably by different bacterial strains and antimicrobial assays used, these results correspond well with our findings. Regarding cytotoxicity, the current study reports that *C. citratus* EO is moderately toxic (IC_50_ = 19.63 µg/mL) to the lung fibroblasts. A previous study reported a similar mild cytotoxic effect on human fibroblast cell line WI38 with an IC_50_ value of 49.39 μg/mL [29]. The minor variance in the IC_50_ values can be attributed to the modification of the cytotoxicity assay previously recommended for evaluation of biological properties of the volatile agents. The use of EVA Capmat protects microtiter plates against vapor transition and provides more reliable results [49]. The safety of *C. citratus* EO for potential inhalation use can be supported by a previously published study reporting a nontoxic effect of vapors of its predominant compound citral on Sprague–Dawley rats at concentrations up to 34 ppm [50]. Furthermore, other published data on the in vivo acute oral toxicity of *C. citratus* EO in mice and rabbit models have been reported to be nontoxic with an LD_50_ value > 2000 mg/kg [51]. Although *C. citratus* EO is classified as GRAS, further toxicological evaluation is necessary to confirm its nontoxicity for practical application in inhalation therapy. GC/MS analysis showed that the major constituents of *C. citratus* EO were *α*- and *β*-citral. These findings are consistent with those of several previously published studies. For instance, El-Kased and El-Kersh [52] and Hanaa et al. [53] reported α-citral (36.35 and 34.98%) and β-citral (34.99 and 40.72%) as the main components of hydrodistilled EO from this species. Citral has also been identified as the main antimicrobial compound of *C. citratus* EO [54].

The EO from rhizomes of *C. scariosus* is used as an ingredient in several Ayurvedic formulations as an anti-infective agent [55]. Previous studies have reported the growth-inhibitory effect of *C. scariosus* EO against carbapenem-resistant *Klebsiella pneumoniae* and methicillin-resistant *S. aureus* in liquid media [56]. In our study, we have observed only weak antibacterial activity of the EO. Considering cytotoxicity, the present research reports that *C. scariosus* EO is nontoxic to the human lung cells. According to our best knowledge, there are no studies reporting the toxicity of the EO from this species. In correspondence with results of our study, cyperene (20.1%) and cyperotundone (10.30%) have previously been identified as abundant components of *C. scariosus* EO [57]. Since a significant variability of chemical composition has been reported for *C. scariosus* EO extracted from plants grown in different geographical locations in India (e.g., content of caryophyllene oxide varied from 2.42 to 10.38%) [58], the geographical origin of plant material analyzed in this study can explain minor differences in the concentrations of detected compounds.

Despite the recent progress in evaluation of biological properties of volatile agents, development of new methodologies suitable for determination of antimicrobial effects in the vapor phase remains a challenge [27]. Recently, a broth macrodilution volatilization assay was developed by our team for evaluation of antimicrobial activity of volatile agents in liquid and vapor phases [59], which combines the principles of broth microdilution volatilization [13] and standard macrodilution methods [60]. The method is performed in commercially available microtubes, which can be tightly closed with a snap cap preventing loss of the active agents by evaporation. Another advantage is that appropriate amounts of media suitable for the cultivation of a broad spectrum of microorganisms (including slow growing fungi) can be applied in microtubes and their caps. However, our previous study assessed only several representatives of volatile phytochemicals. The results of experiments described in this paper clearly demonstrate the validity of a new assay for testing the susceptibility of bacterial pathogens causing respiratory infections to EOs and their vapors.

## 4. Materials and Methods

### 4.1. Chemicals

In biological assays, dimethyl sulfoxide (DMSO, CAS 67-68-5) and MTT dye (CAS 298-93-1) were used as solvent and indicator of cell viability, respectively. Amoxicillin (90%, CAS 26787-78-0), ampicillin (84.5%, CAS 69-52-3), oxacillin (86.3%, CAS 7240-38-2), and tetracycline (98–102%, CAS 60-54-8) were assayed as positive controls. Chemical standards, namely 3-carene (99%, CAS: 498-15-7), camphene (97.5%, CAS: 79-92-5), caryophyllene oxide (99%, CAS: 1139-30-6), citral (95%, CAS: 5392-40-5), linalool (97%, CAS: 78-70-6), *m*-cymene (99%, CAS: 535-77-3), thymol (99%, CAS: 89-83-8), *α*-pinene (99%, CAS: 7785-70-8), β-pinene (99.0%, CAS: 18172-67-3), *γ*-terpinene (97%, CAS: 99-85-4), and *n*-alkanes were used for GC-MS analyses. With the exception of *n*-hexane (CAS: 110-54-3) obtained from (Merck KGaA, Darmstadt, Germany), all other chemicals were purchased from Sigma-Aldrich (Prague, Czech Republic).

### 4.2. Plant Material and Sample Preparation

Plant materials were purchased from Bhagyashree Herbal Farms (Chhattisgarh, India). Dried aerial parts of *C. citratus*, rhizomes of *C. scariosus*, and seeds of *T. ammi* were homogenized using a Grindomix apparatus (GM 100 Retsch, Haan, Germany). The residual moisture contents of both samples were determined gravimetrically at 130 °C for 1 h by a Scaltec SMO 01 analyzer (Scaltec Instruments, Gottingen, Germany) in triplicate according to the Official Methods of Analysis of the Association of Official Agricultural Chemists [61]. EOs were extracted by hydrodistillation of 100 g of ground plant materials in 1 L of distilled water for 3 h using a Clevenger-type apparatus (Merci, Brno, Czech Republic) according to the procedure described in the European Pharmacopeia (2013) [62] and stored in sealed glass vials at +4 °C.

### 4.3. Bacterial Strains and Culture Media

The following four bacterial standard strains from the American Type Culture Collection (ATCC, Manassas, VA, USA) were used: *H. influenzae* ATCC 49247, *S. aureus* ATCC 29213, *S. pneumoniae* ATCC 49619, and *S. pyogenes* ATCC 19615. Cultivation and assay media (broth/agar) were Mueller–Hinton broth (MHB) complemented by Haemophilus test medium (*H. influenzae*), MHB (*S. aureus*), and brain–heart infusion (*S. pneumoniae* and *S. pyogenes*). The pH of the broths was adjusted to a final value of 7.6 using Trizma base (Sigma-Aldrich). All microbial strains and cultivation media were purchased from Oxoid (Basingstoke, UK). Stock cultures of bacterial strains were cultivated in broth medium at 37 °C for 24 h prior to testing in the incubator (Memmert GmbH & Co. KG, Buchenbach, Germany). For the preparation of inoculum, the turbidity of the bacterial suspension was adjusted to 0.5 McFarland standard using a Densi-La-Meter II (Lachema, Brno, Czech Republic) to obtain a final concentration of 10^8^ colony-forming units per mL.

### 4.4. Antimicrobial Assay

EOs were tested for their in vitro antibacterial activity using the broth macrodilution volatilization method at various concentrations in the liquid and vapor phases [59] in standard 2 mL microtubes with snap covers (Eppendorf, Hamburg, Germany). Each EO was dissolved in DMSO, which was subsequently diluted in the appropriate broth medium. To make sufficient stock solutions and to prevent the loss of active chemicals by evaporation, six twofold serially diluted concentrations of samples were created in 15 mL test tubes (Gama Group, Ceske Budejovice, Czech Republic). The starting concentration of tested EOs was 1024 µg/mL. In the subsequent phase, 90 mL of melted agar was pipetted into the rims of the caps, and when the agar had solidified, 5 mL of bacterial suspension was added. Then, with a final volume of 1500 µL, the proper concentrations of each sample that had been prepared in test tubes were pipetted into microtubes. The microtubes were then sealed properly after being injected with 10 µL of bacterial solution. As growth and purity controls, microtubes with inoculated and non-inoculated medium were prepared, respectively. The experiment was incubated at 37 °C for 24 h. Thereafter, the MICs were determined by visual assessment of bacterial growth after coloring the metabolically active bacterial colonies with MTT dye. The respective volumes of 30 and 375 µL of MTT at a concentration of 600 µg/mL were pipetted into the caps and in the microtubes when the interface of color change from yellow to purple (relative to that of colors in control wells) was recorded in broth and agar. The MIC values were determined as the lowest concentrations that inhibited bacterial growth compared with the compound-free control, and they were expressed in µg/mL. DMSO, assayed as the negative control, did not inhibit any of the strains at the concentrations used in the assay (≤1%). The respective susceptibilities of *H. influenzae*, *S. aureus*, *S. pneumoniae*, and *S. pyogenes* to ampicillin, oxacillin, amoxicillin, and tetracycline were taken as positive antibiotic controls [63]. All tests were performed as three independent experiments, each carried out in triplicate, and the results were presented as median/modal values. According to the widely accepted norm in MIC testing, the mode and median were used for the final value calculation when triplicate endpoints were within the two- and three-dilution range, respectively.

### 4.5. Cell Cultures

Lung fibroblast cell line MRC-5, obtained from ATCC (Manassas, VA, USA), was propagated in Eagle’s minimum essential medium (EMEM) supplemented with 10% fetal bovine serum (FBS), 2 mM glutamine, 10 µL/mL nonessential amino acids, and 1% penicillin–streptomycin solution (10,000 units/mL of penicillin and 10 mg/mL of streptomycin); all these components were purchased from Sigma-Aldrich. The cells were pre-incubated in 96-well microtiter plates at a density of 2.5 × 10^3^ cells per well for 24 h at 37 °C in a humidified incubator (Sanyo Electric Co, Ltd., Osaka, Japan) in an atmosphere of 5% CO_2_ in air. Vinorelbine (≥98%, CAS 125317-39-7), used as positive control, was also obtained from Sigma-Aldrich.

### 4.6. Cytotoxicity Assay

The modified MTT test, which is based on the metabolism of MTT to blue formazan by mitochondrial dehydrogenases in living lung cells, was used in the current investigation [64]. The evaluated EOs samples were applied to MRC-5 lung fibroblast cells grown in 96-well plates for 72 h. EOs were diluted in EMEM medium supplemented with 10% FBS after being dissolved in DMSO at a maximum concentration of 1%. The samples were then serially diluted eight times, yielding concentrations ranging from 256 to 2 µg/mL. The microtiter plates were incubated for 72 h at 37 °C in a humidified atmosphere of 5% CO_2_ in air with ethylene vinyl acetate EVA Capmats (USA Scientific Inc., Ocala, FL, USA). After that, MTT reagent (1 mg/mL) in EMEM solution was added to each well and the plates were incubated for an additional 2 h at 37 °C in a humidified atmosphere of 5% CO_2_ in air. Once the medium had been removed from the incubation, the intracellular formazan product had been dissolved in 100 µL of DMSO. The viability of the lung cells at the tested concentration (1%) was unaffected by the solvent utilized. The absorbance was measured at 555 nm using a Tecan Infinite M200 spectrometer (Tecan Group, Mannedorf, Switzerland), and the viability was computed in comparison to the untreated control. For each test, three independent experiments, each with two replicates, were conducted. Using the GraphPad Prism program (GraphPad Software, La Jolla, CA, USA), the results of the cytotoxicity effect were calculated and expressed as average IC_50_ with standard deviation in µg/mL. According to the Special Programme for Research and Training in Tropical Diseases (WHO-Tropical Diseases) [30], the levels of cytotoxic effects were categorized as cytotoxic (IC_50_ < 2 µg/mL), moderately cytotoxic (IC_50_ 2–89 µg/mL), and nontoxic (IC_50_ > 90 µg/mL). Furthermore, for the purpose of comparing microbiological and toxicological data, IC_80_ was determined to be equal to the MIC endpoint [65]. To compare the quantity of toxic antibacterial agents with the amount of effective antibacterial agents, therapeutic indices (TIs) were defined as the ratio of IC_80_ and x-MIC values [66].

### 4.7. Chemical Analysis of EOs’ Liquid Phase

The dual-column/dual-detector gas chromatograph Agilent GC-7890B system (Agilent Technologies, Santa Clara, CA, USA) equipped with autosampler Agilent 7693, two columns, a fused-silica HP-5MS (30 m × 0.25 mm, film thick-ness 0.25 µm, Agilent 19091s-433), a DB-Heavy WAX (30 m × 0.25 mm, film thickness 0.25 µm, Agilent 122–7132), and a flame ionization detector (FID) coupled with single quadrupole mass selective detector Agilent MSD-5977B were used to characterize the chemical composition of the targeted EOs. Helium was used as the carrier gas at a rate of 1 mL/min, and the injector temperature for both columns was 250 °C. After 3 min, the oven temperature for both columns increased from 50 to 280 °C. Initially, the heating velocity was 3 °C/min until the system reached a temperature of 120 °C. Subsequently, the velocity increased to 5 °C/min until a temperature of 250 °C, and after 5 min holding time, the heating speed reached 15 °C/min until obtaining a temperature of 280 °C. Heating was followed by an isothermic period of 20 min. The EO samples were diluted in *n*-hexane for GC/MS at a concentration of 20 µL/mL. One microliter of the solution was injected in split mode in a split ratio of 1:30. The mass detector was set to the following conditions: ionization energy 70 eV, ion source temperature 230 °C, scan time 1 s, and mass range 40–600 *m*/*z*. Identification of constituents was based on a comparison of their retention indices (RI) and retention times (RT) and spectra with the National Institute of Standards and Technology Library ver. 2.0.f (NIST, Gaithersburg, MD, USA) [33], as well as with authentic standards. The RIs were calculated for compounds separated by the HP-5MS column using the retention times of *n*-alkanes series ranging from C_8_ to C_40_. For each EO analyzed, the final number of compounds was calculated as the sum of components simultaneously identified using both columns and the remaining constituents identified by individual columns only. Quantitative data were expressed as relative percentage content of constituents determined by the FID.

### 4.8. Chemical Analysis of EOs’ Vapor Phase

For the chemical analysis of EOs’ vapors, *T. ammi* was chosen, as it exhibited the highest antimicrobial potential. Similarly, like Antih et al. [43], two distinct sampling methods—HS-SPME and HS-GTS—were used to sample the headspace above a solution of MHB and EO at a concentration of 256 µg/mL, which was observed as the lowest MIC value of this EO in vapor phase. Five samples were made for each experiment (one for every three hours during the 12 h incubation period), and a volume of 2 mL of the combination was added to a 4 mL glass vial. Except for the first sample (t = 0 h), all the samples were incubated in the same way as the bacterial cultures at a temperature of 37 °C until they were analyzed at 3, 6, and 12 h. For the HS-SPME, a fiber assembly coated with a 50/30 m mixed layer of divinylbenzene/carboxen/polydimehylsiloxane (DVB/CAR/PDMS—SUPELCO, Bellefonte, PA, USA) was used. The coated fiber was exposed to the headspace for 15 min to allow the adsorption of the volatile chemicals above the mixture (EO and MHB) until the headspace had reached equilibrium. The fiber was then retained in the injector for the duration of the analysis until the next measurement, when the needle was removed and put into the GC injector port. The injection method was set at splitless mode and injector temperature was set at 250 °C. For the HS-GTS sampling technique, a 2.5 mL SampleLock gas-tight syringe (Hamilton Bonaduz AG, Bonaduz, Switzerland) was used, which has a twist valve lock and a positive rear plunger stop to prevent sample loss. At equilibrium, the needle was passed through the vial septum and inserted until reaching the middle of the headspace, and after collecting the headspace, the valve of the syringe was closed. Afterward, the syringe was removed from the vial and inserted into the GC injector at a similar temperature of 250 °C, but the injection mode was set as spitless mode. For both sampling methods, measurements were repeated every 3 h during a 12 h incubation period. Furthermore, analysis was performed on the HP-5MS column with similar operational parameters as described earlier for GC/MS analysis.

The chemical analysis of the all EO samples was performed in triplicate, including the chromatographic analysis of its liquid phase and the headspace analysis using both extraction techniques (HS-SPME and HS-GTS). Relative peak area percentages were expressed as mean average of these three measurements ± standard deviation.

## 5. Conclusions

In summary, this study reports validation of the broth macrodilution volatilization method for assessment of antibacterial properties of EOs and shows in vitro growth-inhibitory effect of EOs hydrodistilled from three Indian medicinal plants, namely *C. citratus*, *C. scariosus*, and *T. ammi*, against pneumonia-causing bacteria *H. influenzae*, *S. aureus*, *S. pneumoniae*, and *S. pyogenes* in liquid and vapor phase. Among all EOs tested, the strongest antibacterial effect was observed for *T. ammi* EO against *H. influenzae* with MIC 128 and 256 µg/mL in the liquid and vapor phases. The results of the cytotoxicity testing showed no toxicity of *C. scariosus* EO to the normal lung fibroblasts. The GC/MS analysis identified *α*-citral, cyperotundone, and thymol as the main constituents of *C. citratus*, *C. scariosus*, and *T. ammi* EOs, respectively. In a series of headspace experiments, β-cymene (52–49%), γ-terpinene (39–35%), thymol (12–2%), and β-pinene (6–2%) were identified as the major compounds of *T. ammi* EO vapors when analyzed using HS-GTS and HS-SPME sampling techniques. These compounds can be associated with imparting an antibacterial effect in the vapor phase. Furthermore, HS-SPME and HS-GTS have been proven to be complementary methods suitable for studying the qualitative and quantitative aspects of the volatile profiles of EO, subsequently. Overall, the broth macrodilution volatilization method can be recommended for testing the susceptibility of bacteria to EOs. In addition, *T. ammi* EO seems to be a promising antibacterial agent for further research in the area of inhalation therapies. However, further experiments focused on the safety and in vivo efficacy of EOs analyzed in this study are required to verify their practical applicability.

## Figures and Tables

**Table 1:** In vitro growth-inhibitory effect of Indian essential oils in the liquid and vapor phases against respiratory pathogens.

Sample	Bacterium/Growth Medium/Minimum Inhibitory Concentration (µg/mL)								
Essential Oil	*Haemophilus influenzae*		*Staphylococcus aureus*		*Streptococcus pneumoniae*		*Streptococcus pyogenes*		x-MIC
	Broth	Agar	Broth	Agar	Broth	Agar	Broth	Agar	
*Cymbopogon citratus*	256	256	512	1024	512	1024	512	1024	448
*Cyperus scariosus*.	1024	1024	1024	>1024	1024	>1024	1024	>1024	1024
*Trachyspermum ammi*	128	256	512	512	512	1024	512	1024	416
**Positive antibiotic control**	1 ^a^	n.d.	0.5 ^b^	n.d.	0.25 ^c^	n.d.	0.25 ^d^	n.d.	-

x-MIC: mean value of minimal inhibitory concentrations in broth medium; positive antibiotic control: ^a^ ampicillin, ^b^ oxacillin, ^c^ amoxicillin, ^d^ tetracycline; n.d.: not detected.

**Table 2:** Cytotoxicity of Indian essential oils to the normal lung fibroblast cell line MRC-5.

Sample	IC_50_ ± SD (µg/mL)	IC_80_ ± SD (µg/mL)	TI
**Essential oil**			
*Cymbopogon citratus*	19.63 ± 1.02	29.54 ± 2.18	0.065
*Cyperus scariosus*	>258	>258	>0.252
*Trachyspermum ammi*	82.04 ± 3.39	156.57 ± 13.88	0.376
**Positive control**			
vinorelbine	0.54 ± 0.26	>10	n.a.

IC_50_: half maximal inhibitory concentration of proliferation in μg/mL; IC_80_: 80% inhibitory concentration of proliferation in μg/mL; SD: standard deviation; TI: therapeutic index (TI = IC_80_/x-MIC); n.a.: not applicable.

**Table 3:** Chemical composition of *Cymbopogon citratus* essential oil.

RI ^a^		Compound	Cl. ^b^	Column			
				Content ^c^ (%)		Identification ^f^	
Obs.	Lit.			HP-5MS	DB-WAX	HP-5MS	DB-WAX
915	926	Tricyclene	MH	0.26 ± 0.12	tr. ^d^	RI, MS, Std,	MS
927	939	α-Pinene	MH	0.20 ± 0.04	- ^e^	RI, MS, Std	-
942	953	Camphene	MH	2.42 ± 0.79	1.62 ± 0.34	RI, MS, Std	MS
979	985	Sulcatone	MO	0.35 ± 0.18	-	RI, MS	-
1023	1029	*D*-Limonene	MH	0.23 ± 0.03	-	RI, MS	-
1029	1050	*trans*-β-Ocimene	MH	0.31 ± 0.09	-	RI, MS	-
1039	1011	3-Carene	MH	0.13 ± 0.02	-	RI, MS, Std	-
1063	1030	4-Nonanone	MO	1.41 ± 0.38	1.55 ± 0.97	RI, MS	MS
1098	1098	Linalool	MO	0.46 ± 0.24	0.31 ± 0.07	RI, MS, Std	MS
1178	1184.7	Isogeranial	MO	0.70 ± 0.01	0.55 ± 0.05	RI, MS	MS
1190	1189	α-Terpineol	MO	0.39 ± 0.03	-	RI, MS	-
1238	1240	β-Citral	MO	35.8 ± 0.61	24.3 ± 8.82	RI, MS, Std	MS
1268	1270	α-Citral	MO	48.9 ± 0.55	33.2 ± 11.2	RI, MS, Std	MS
1376	1381	Geranyl acetate	MO	2.02 ± 0.20	tr.	RI, MS	MS
1412	1418	Caryophyllene	SH	0.45 ± 0.31	0.75 ± 0.05	RI, MS	MS
1510	1513	γ-Cadinene	SH	1.19 ± 0.33	1.20 ± 0.23	RI, MS	MS
^g^	1797	Geraniol	MO	-	0.77 ± 0.07	-	MS
1582	1581	Caryophyllene oxide	SH	3.0 ± 1.34	2.88 ± 1.40	RI, MS, Std	MS
^g^	1430	α-Cyclocitral	MO	-	0.45 ± 0.04	-	MS
^g^	NA	Isoneral	MO	-	0.36 ± 0.02	-	MS
^g^	1669	Isoborneol	MO	-	0.83 ± 0.01	-	MS
**Total content (%)**				**99.62**	**68.** **77**		

^a^ RI = retention indices; Obs. = retention indices determined relative to a homologous series of *n*-alkanes (C_8_–C_40_) on an HP-5MS column; Lit. = literature RI values [31,32], NA = RI values not available in the literature; ^b^ Cl = class; MH—monoterpene hydrocarbons, MO—oxygenated monoterpenoids, SH—sesquiterpene hydrocarbons; ^c^ relative peak area percentage as mean of three measurements ± standard deviation; ^d^ tr. = traces, relative peak area < 0.05%; ^e^ - = not detected; ^f^ identification method: MS = mass spectrum was identical to that of the National Institute of Standards and Technology Library (ver. 2.0.f), RI = the retention index was matching literature database; Std = constituent identity confirmed by co-injection of authentic standards; ^g^ retention indices were not calculated for compounds determined by DB-WAX column.

**Table 4:** Chemical composition of *Cyperus scariosus* essential oil.

RI ^a^		Compound	Cl. ^b^	Column			
				Content ^c^ (%)		Identification ^f^	
Obs.	Lit.			HP-5MS	DB-WAX	HP-5MS	DB-WAX
927	939	α-Pinene	MH	1.34 ± 0.10	0.50 ± 0.01	RI, MS, Std	MS
970	980	β-Pinene	MH	1.88 ± 0.33	0.06 ± 0.00	RI, MS, Std	MS
1025	1032	Eucalyptol	MO	0.24 ± 0.16	- ^d^	RI, MS	-
1137	1137	Pinocarveol	MO	1.85 ± 0.23	0.60 ± 0.01	RI, MS	MS
1158	1165	Pinocarvone	MO	0.37 ± 0.32	0.09 ± 0.00	RI, MS	MS
1168	1193	Myrtenal	MO	0.41 ± 0.27	0.13 ± 0.30	RI, MS	MS
1314	1327	Cyprotene	SH	0.16 ± 0.05	tr.^e^	RI, MS	MS
1344	1349	α-Terpinyl acetate	MO	1.65 ± 0.18	tr.	RI, MS	MS
1371	1376	Copaene	SH	1.46 ± 0.47	tr.	RI, MS	MS
1394	1398	Cyperene	SH	9.87 ± 0.59	8.5 ± 0.04	RI, MS	MS
1446	1477	α-Muurolene	SH	0.16 ± 0.04	tr.	RI, MS	MS
1456	1461	Rotundene	SH	1.94 ± 0.07	1.25 ± 0.01	RI, MS	MS
1483	1473.7	γ-Patchoulene	SH	0.19 ± 0.05	tr.	RI, MS	MS
1489	1491	Valencene	SH	0.63 ± 0.08	0.57 ± 0.60	RI, MS	MS
1518	1518	β-Cadinene	SH	0.31 ± 0.18	0.08 ± 0.40	RI, MS	MS
1528	1532	Cyperene epoxide	SO	2.65 ± 0.26	1.50 ± 0.00	RI, MS	MS
1541	1542	α-Calacorene	SH	0.13 ± 0.05	-	RI, MS	-
1565	1579	Isoaromadendrene epoxide	SO	0.62 ± 0.07	1.03 ± 0.00	RI, MS	MS
1572	1627	Longiverbenone	SO	1.33 ± 0.15	1.20 ± 0.08	RI, MS	MS
1582	1581	Caryophyllene oxide	SH	19.79 ± 0.58	17.54 ± 0.12	RI, MS, Std	MS
1591	NA	β-Santalol	SO	0.38 ± 0.12	-	RI, MS	-
1609	1608	Humulene epoxide 2	SO	1.69 ± 0.25	2.60 ± 0.10	RI, MS	MS
1656	1604	Globulol	SO	0.23 ± 0.04	0.39 ± 0.03	RI, MS	MS
1664	1663	Patchouli alcohol	SO	0.50 ± 0.05	-	RI, MS	-
1677	1676	Mustakone	SO	6.26 ± 0.26	3.67 ± 0.30	RI, MS	MS
1697	1694	Cyperotundone	SO	29.1 ± 1.11	28.91 ± 0.72	RI, MS	MS
1750	1752	Aristolone	SO	3.17 ± 0.77	3.73 ± 0.10	RI, MS	MS
1808	1807	Nootkatone	SO	2.17 ± 0.49	2.03 ± 0.40	RI, MS	MS
^g^	NA	β-Pinone	MO	-	tr.	-	MS
^g^	1586	β-Elemene	SH	-	tr.	-	MS
^g^	1652	*cis*-Verbenol	MO	-	tr.	-	MS
^g^	NA	Aristolochene	SH	-	tr.	-	MS
^g^	1680	α-Terpineol			tr.	-	MS
^g^	NA	α-Maaliene	SH	-	tr.	-	MS
^g^	1784	Myrtenol	MO	-	tr.	-	MS
^g^	2063	Cubenol	SO	-	0.49 ± 0.00	-	MS
^g^	1978	α-Cedrene epoxide	SO	-	0.40 ± 0.10	-	MS
^g^	NA	Aromadendrene oxide-(1)	SO	-	1.35 ± 0.40	-	MS
^g^	NA	Calarene epoxide	SO	-	0.52 ± 0.02	-	MS
^g^	NA	Diepicedrene-1-oxide	SO	-	0.05 ± 0.00	-	MS
**Total content (%)**				**91.48**	**77.17**		

^a^ RI = retention indices; Obs. = retention indices determined relative to a homologous series of *n*-alkanes (C_8_–C_40_) on an HP-5MS column; Lit. = literature RI values [31,32], NA = RI values not available in the literature; ^b^ Cl = class; MH—monoterpene hydrocarbons, MO—oxygenated monoterpenoids, SH—sesquiterpene hydrocarbons, SO—oxygenated sesquiterpenoids; ^c^ relative peak area percentage as mean of three measurements ± standard deviation; ^d^ - = not detected; ^e^ tr. = traces, relative peak area < 0.05%; ^f^ identification method: MS = mass spectrum was identical to that of the National Institute of Standards and Technology Library (ver. 2.0.f), RI = the retention index was matching literature database; Std = constituent identity confirmed by co-injection of authentic standards; ^g^ retention indices were not calculated for compounds determined by DB-WAX column.

**Table 5:** Chemical composition of *Trachyspermum ammi* essential oil.

RI ^a^		Compound	Cl. ^b^	Column			
				Content ^c^ (%)		Identification ^e^	
Obs.	Lit.			HP-5MS	DB-WAX	HP-5MS	DB-WAX
920	917	β-Thujene	MH	0.30 ± 0.03	0.17 ± 0.02	RI, MS	MS
964	925	α-Pinene	MH	0.19 ± 0.01	0.42 ± 0.13	RI, MS, Std	MS
981	971	β-Pinene	MH	1.87 ± 0.25	2.13 ± 0.62	RI, MS, Std	MS
994	984	β-Myrcene	MH	0.21 ± 0.06	0.38 ± 0.03	RI, MS	MS
1007	974	2-Carene	MH	0.20 ± 0.02	- ^d^	RI, MS	MS
1050	1031	β-Cymene	MH	22.6 ± 0.89	17.1 ± 3.99	RI, MS, Std	MS
1079	1065	γ-Terpinene	MH	21.5 ± 0.86	17.6 ± 0.99	RI, MS, Std	MS
1175	1086	Isoterpinolene	MH	0.08 ± 0.06	-	RI, MS	MS
1315	1290	Thymol	MO	51.2 ± 1.25	45.8 ± 4.41	RI, MS, Std	MS
^f^	1172	α-Terpinene	MH	-	0.12 ± 0.01	-	MS
^f^	1244	*D*-Limonene	MH	-	0.10 ± 0.01	-	MS
^f^	1195	β-Phellandrene	MH	-	0.08 ± 0.03	-	MS
^f^	NA	*trans*-2-Caren-4-ol	MO	-	0.18 ± 0.02	-	MS
^f^	1680	Terpineol	MO	-	0.06 ± 0.01	-	MS
^f^	1635	Terpinen-4-ol	MO	-	0.20 ± 0.04	-	MS
**Total content (%)**				**99.47**	**84.26**		

^a^ RI = retention indices; Obs. = retention indices determined relative to a homologous series of *n*-alkanes (C_8_–C_40_) on an HP-5MS column; Lit. = literature RI values [31,32], NA = RI values not available in the literature; ^b^ Cl = class; MH—monoterpene hydrocarbons, MO—oxygenated monoterpenoids, SH—sesquiterpene hydrocarbons; ^c^ relative peak area percentage as mean of three measurements ± standard deviation; ^d^ - = not detected; ^e^ identification method: MS = mass spectrum was identical to that of the National Institute of Standards and Technology Library (ver. 2.0.f), RI = the retention index was matching literature database; Std = constituent identity confirmed by co-injection of authentic standards; ^f^ retention indices were not calculated for compounds determined by DB-WAX column.

**Table 6:** Chemical composition of a headspace above a *Trachyspermum ammi* essential oil dissolved in Mueller–Hinton broth at a concentration of 256 µg/mL.

RI ^a^		Compound	Extraction Method/Time (h)/Content ^b^ (%)										Ident. ^e^
			Solid Phase Microextraction					Gas Tight Syringe Extraction					
Obs.	Lit.		0	3	6	9	12	0	3	6	9	12	
925	939	α-Pinene	1.27 ± 0.10	1.46 ± 0.00	1.42 ± 0.01	1.41 ± 0.00	1.25 ± 0.01	tr. ^c^	tr.	tr.	tr.	tr.	RI, MS
961	1011	2-Carene	0.64 ± 0.00	0.44 ± 0.00	0.62 ± 0.01	0.65 ± 0.02	0.72. ±0.01	- ^d^	-	-	-	-	RI, MS
971	980	β-Pinene	1.91 ± 0.01	2.80 ± 0.05	2.36 ± 0.00	2.31 ± 0.01	1.79 ± 0.20	6.57 ± 0.20	4.93 ± 0.40	3.89 ± 0.10	4.11 ± 0.01	2.02 ± 0.30	RI, MS
1031	1030	β-Cymene	49.14 ± 1.00	48.00 ± 1.10	46.57 ± 0.80	45.97 ± 1.80	43.17 ± 0.90	52.00 ± 3.50	49.18 ± 2.90	48.67 ± 2.50	46.32 ± 1.40	45.60 ± 0.60	RI, MS
1065	1062	γ-Terpinene	39.36 ± 0.70	35.41 ± 0.50	35.11 ± 0.90	33.26 ± 0.60	31.01 ± 0.20	35.00 ± 2.50	32.23 ± 0.09	31.60 ± 0.50	32.22 ± 1.30	28.20 ± 2.90	RI, MS
1306	1290	Thymol	4.96 ± 0.01	5.66 ± 0.40	9.82 ± 0.08	11.90 ± 0.93	12.10 ± 0.80	tr.	1.23 ± 0.10	2.21 ± 0.20	2.07 ± 0.03	tr.	RI, MS
**Total content (%)**			**97.25**	**94.21**	**95.9**	**95.5**	**90.66**	**93.40**	**88.** **57**	**87.** **98**	**85.72**	**79.** **05**	

^a^ RI = retention indices; Obs. = retention indices determined relative to a homologous series of *n*-alkanes (C_8_–C_40_) on an HP-5MS column; Lit. = literature RI values [31,32]; ^b^ relative peak area percentage as mean of three measurements ± standard deviation; ^c^ tr. = traces, relative peak area < 0.05%; ^d^ - = not detected; ^e^ identification method: MS = mass spectrum was identical to that of the National Institute of Standards and Technology Library (ver. 2.0.f), RI = the retention index was matching literature database.

## Data Availability

Not applicable.

## Sample Availability

Not available.
